# Biallelic *UFM1* and *UFC1* mutations expand the essential role of ufmylation in brain development

**DOI:** 10.1093/brain/awy135

**Published:** 2018-06-02

**Authors:** Michael S Nahorski, Sateesh Maddirevula, Ryosuke Ishimura, Saud Alsahli, Angela F Brady, Anaïs Begemann, Tsunehiro Mizushima, Francisco J Guzmán-Vega, Miki Obata, Yoshinobu Ichimura, Hessa S Alsaif, Shams Anazi, Niema Ibrahim, Firdous Abdulwahab, Mais Hashem, Dorota Monies, Mohamed Abouelhoda, Brian F Meyer, Majid Alfadhel, Wafa Eyaid, Markus Zweier, Katharina Steindl, Anita Rauch, Stefan T Arold, C Geoffrey Woods, Masaaki Komatsu, Fowzan S Alkuraya

**Affiliations:** 1 Cambridge Institute for Medical Research, Wellcome Trust MRC Building Addenbrookes Hospital, Hills Rd, Cambridge, UK; 2 Department of Genetics, King Faisal Specialist Hospital and Research Center, Riyadh, Saudi Arabia; 3 Department of Biochemistry, Niigata University Graduate School of Medical and Dental Sciences, Chuo-ku, Niigata, Japan; 4 North West Thames Genetics Service, Level 8V, St Mark’s Hospital, Northwick Park Hospital Watford Road, Harrow, UK; 5 Institute of Medical Genetics, University of Zurich, 8952 Schlieren-Zurich, Switzerland; 6 Picobiology Institute, Graduate School of Life Science, University of Hyogo, Ako-gun, Hyogo, Japan; 7 King Abdullah University of Science and Technology, Computational Bioscience Research Center, Division of Biological and Environmental Sciences and Engineering, Thuwal, Saudi Arabia; 8 Saudi Human Genome Program, King Abdulaziz City for Science and Technology, Riyadh, Saudi Arabia; 9 King Abdullah International Medical Research Centre, King Saud bin Abdulaziz University for Health Sciences, Division of Genetics, Department of Pediatrics, King Abdullah Specialized Children Hospital, King Abdulaziz Medical City, Ministry of National Guard-Health Affairs (NGHA), Riyadh, Saudi Arabia; 10 Neuroscience Center Zurich, University of Zurich and ETH Zurich, Zurich, Switzerland; 11 Department of Anatomy and Cell Biology, College of Medicine, Alfaisal University, Riyadh, Saudi Arabia

**Keywords:** ufmylation, UFM1, UFC1, encephalopathy, epilepsy

## Abstract

The post-translational modification of proteins through the addition of UFM1, also known as ufmylation, plays a critical developmental role as revealed by studies in animal models. The recent finding that biallelic mutations in *UBA5* (the E1-like enzyme for ufmylation) cause severe early-onset encephalopathy with progressive microcephaly implicates ufmylation in human brain development. More recently, a homozygous *UFM1* variant was proposed as a candidate aetiology of severe early-onset encephalopathy with progressive microcephaly. Here, we establish a locus for severe early-onset encephalopathy with progressive microcephaly based on two families, and map the phenotype to a novel homozygous *UFM1* mutation. This mutation has a significantly diminished capacity to form thioester intermediates with UBA5 and with UFC1 (the E2-like enzyme for ufmylation), with resulting impaired ufmylation of cellular proteins. Remarkably, in four additional families where eight children have severe early-onset encephalopathy with progressive microcephaly, we identified two biallelic *UFC1* mutations, which impair UFM1-UFC1 intermediate formation with resulting widespread reduction of cellular ufmylation, a pattern similar to that observed with *UFM1* mutation. The striking resemblance between *UFM1*- and *UFC1*-related clinical phenotype and biochemical derangements strongly argues for an essential role for ufmylation in human brain development. The hypomorphic nature of *UFM1* and *UFC1* mutations and the conspicuous depletion of biallelic null mutations in the components of this pathway in human genome databases suggest that it is necessary for embryonic survival, which is consistent with the embryonic lethal nature of knockout models for the orthologous genes.

## Teaser

Ufmylation is the post-translational modification of proteins through the addition of UFM1. Nahorksi *et al.* identify mutations in *UFM1* and in *UFC1*, which encodes an enzyme required for ufmylation, in individuals with severe early-onset encephalopathy with progressive microcephaly. The findings suggest an essential role for ufmylation in human brain development.

## Introduction

Post-translational modification greatly expands the functional repertoire of proteins beyond the confines of their coding sequence, and serves a diverse array of regulatory roles in protein turnover, localization and interactions (Hitosugi and Chen, 2014; Tompa *et al.*, 2014). Ubiquitination is a well-established post-translational modification involving a series of enzymatic reactions that add ubiquitin to its target protein (Hershko and Ciechanover, 1998). These reactions involve a few E1 activating enzymes, dozens of E2 conjugating enzymes and hundreds of E3 ligating enzymes. More than a dozen ubiquitin-like (UBL) proteins have been found to similarly modify their target proteins (Schulman and Harper, 2009). UBL are classified into those that are activated and conjugated to substrates (type I) e.g. SUMO, NEDD8, ATG8, ATG12, URM1, UFM1, FAT10, and ISG15, and those that do not undergo conjugation (type II) (Cappadocia and Lima, 2018).

UFM1 (ubiquitin-fold modifier 1), a 9.1-kDa protein with a tertiary structure similar to ubiquitin, is among the most recently identified type I UBL proteins (Komatsu *et al.*, 2004). The process of covalently attaching UFM1 to its target proteins, termed ufmylation, requires E1 activating enzymes (UBA5), E2 conjugating enzymes (UFC1), and an E3 ligase (UFL1) (Daniel and Liebau, 2014). UFM1 is activated by UBA5, forming a high-energy thioester bond. Activated UFM1 is then transferred to UFC1, in a similar thioester linkage (Komatsu *et al.*, 2004). Ufmylation is completed by the catalysis of UFM1 transfer to the substrate protein through the action of the E3 enzyme UFL1 (Tatsumi *et al.*, 2010). The physiological context of ufmylation remains incompletely understood. The literature on the relevance of ufmylation to human health was originally limited to cancer and, to a much lesser extent, complex diseases such as diabetes, ischaemic heart diseases and alcoholic liver disease (Liu *et al.*, 2014; Yoo *et al.*, 2015; Wei and Xu, 2016). As with other biochemical processes, however, Mendelian diseases involving deficiency of the individual components of ufmylation offered a unique opportunity to reveal the extent to which ufmylation influences human physiology. We and others have reported hypomorphic mutations in *UBA5* in children with severe infantile onset epileptic encephalopathy (Colin *et al.*, 2016; Duan *et al.*, 2016; Muona *et al.*, 2016). Additionally, Hamilton *et al.* (2017) reported that a homozygous mutation in *UFM1* caused a severe early-onset encephalopathy with progressive microcephaly, although the mechanism remained unclear. In this report, we provide evidence that it is the process of ufmylation that is essential for normal nervous system development and function, based on the identification of *UFM1* and *UFC1* biallelic mutations, which we show lead to a remarkably similar neurological phenotype and accompanying impairment in ufmylation.

## Materials and methods

### Human subjects

Patients and available relatives were recruited with informed consent as part of IRB-approved research protocols (RAC# 2080006 and 2121053; StV 11/09). Clinical data, including laboratory and imaging studies, were collected from all participants. Blood was collected in EDTA tubes for DNA extraction and in Na-heparin tubes for the establishment of lymphoblastoid cell lines.

### Autozygome analysis

Genomewide single nucleotide polymorphism (SNP) genotyping using Axiom SNP Chip (Affymetrix) from all available patients and relatives was pursued to determine the candidate autozygome as described before (Alkuraya, 2010, 2012). Runs of homozygosity >2 Mb were considered surrogates of autozygosity given the consanguineous nature of the study families as determined by AutoSNPa. Homozygosity mapping was performed on all available family members using HomozygosityMapper (http://www.homozygositymapper.org/).

### Exome sequencing and variant filtering

Exome capture was performed using TruSeq Exome Enrichment kit (Illumina) following the manufacturer’s protocol. Samples were prepared as an Illumina sequencing library, and in the second step, the sequencing libraries were enriched for the desired target using the Illumina Exome Enrichment protocol. The captured libraries were sequenced using Illumina HiSeq 2000 Sequencer. The reads were mapped against UCSC hg19 (http://genome.ucsc.edu/) by BWA (http://bio-bwa.sourceforge.net/). The SNPs and indels were detected by SAMtools (http://samtools.sourceforge.net/). Variants from whole exome sequencing (WES) were filtered such that only novel (or very low frequency <0.1%), coding/splicing, homozygous variants that are within the candidate autozygome (autozygous intervals exclusive to the affected individuals) and are predicted to be pathogenic were considered as likely causal variants (Alkuraya, 2013, 2016). Frequency of variants was determined using publicly available variant databases (1000 Genomes, Exome Variant Server and ExAC) as well as a database of 2369 in-house ethnically-matched exomes. Pathogenicity is likely if the mutation is loss-of-function (splicing/truncating) or, in the case of missense/in-frame indels, removes a highly conserved amino acid and is predicted to be pathogenic by the three *in silico* prediction modules PolyPhen, SIFT and CADD.

### *In silico* modelling

3D experimental structures were retrieved from the Protein Data Bank (PDB) and analysed using PYMOL (www.pymol.org).

### Pull-down assay

Recombinant proteins were purified with glutathione S-transferase (GST) affinity purification system according to the manufacturer’s protocol (GE Healthcare). The GST moiety of all purified proteins without GST-UBA5 was removed on column by PreScission protease (GE Healthcare). GST-UBA5 bound to Glutathione Sepharose® 4B (GE Healthcare) was incubated for 20 min at 4°C with indicated purified proteins in pull-down assay buffer (20 mM Tris-Cl, pH 7.5, 150 mM NaCl, 1 mM EDTA, 1 mM DTT, 0.05% Nonidet P-40). The pulled-down protein complexes were extensively washed with pull-down assay buffer, and the obtained samples were subjected to NuPAGE® (4–12% acrylamide gradient) and Coomassie brilliant blue staining.

### Cell culture

HEK293T cells were grown in Dulbecco’s modified Eagle medium (DMEM) containing 10% foetal bovine serum (FBS), 5 U/ml penicillin, and 50 µg/ml streptomycin. Lymphoblasts were cultured in RPMI 1640 supplemented by 10% FBS, 5 U/ml penicillin, and 50 µg/ml streptomycin. To generate *UFM1*- and *UFC1*-knockout cells, each *UFM1* and *UFC1* guide RNA designed using the CRISPR Design tool (http://crispr.mit.edu/) was subcloned into pX330-U6-Chimeric_BB-CBh-hSpCas9 (Addgene #42230), a human codon-optimized SpCas9 and chimeric guide RNA expression plasmid. HEK293T cells were co-transfected with the pX330 and pEGFP-C1 (#6084-1, Clontech Laboratories) vectors, and cultured for 2 days. Thereafter, the GFP-positive cells were sorted and expanded. Loss of *UFM1* and of *UFC1* was confirmed by heteroduplex mobility assay followed by immunoblot analysis with anti-UFM1 and anti-UFC1 antibodies, respectively.

### Immunoblot analysis

Cells were lysed with ice-cold TNE buffer (10 mM Tris-Cl, pH 7.5, 1% Nonidet P-40, 150 mM NaCl, 1 mM EDTA, and protease inhibitors). The samples were separated using the NuPAGE® system (Invitrogen) on 12% Bis-Tris gels in NuPAGE® MOPS SDS Running Buffer, and transferred to polyvinylidene difluoride (PVDF) membranes. Antibodies against FLAG (Medical & Biological Laboratories Co., Ltd., M185-3L), UFC (Abcam, ab189251) and UFM1 (Abcam, ab109305) were purchased from the indicated suppliers. Anti–UBA5 and UFM1 polyclonal antibodies were described previously (Komatsu *et al.*, 2004). The immunoreactive bands were detected by LAS-4000 (GE Healthcare UK Ltd.). The quantitative densitometric analyses of FLAG-UBA5-MYC-UFM1, FLAG-UFC1-MYC-UFM1, endogenous UBA5-UFM1 intermediate, and endogenous UFC1-UFM1 intermediate relative to free FLAG-UBA5, FLAG-UFC1, endogenous UBA5, and endogenous UFC1 were carried out using Multi Gauge Version 3.2 Image software (Fuji Film, Tokyo, Japan). Statistical analysis was performed using an unpaired *t*-test (Welch test). The data represent the means ± standard error (SE) of three separate experiments.

### *In vitro* thioester formation assay

*In vitro* thioester formation assay was conducted as previously reported (Komatsu *et al.*, 2004). Briefly, recombinant GST-UFM1ΔC2, GST-UFM1ΔC2^R81C^, GST- UFM1^ΔGly83^, GST-UBA5, GST-UFC1, GST-UFC1^R23Q^ and GST-UFC1^T106I^ were produced in *Escherichia coli* and recombinant proteins were purified by chromatography on Glutathione Sepharose® 4B (GE Healthcare). After digestion of GST by PreScission Protease (GE Healthcare), the recombinant proteins were dialyzed against 50 mM BisTris (pH 6.5), 100 mM NaCl, 10 mM MgCl_2_, and 0.1 mM DTT (reaction buffer). Thioester formation reactions contained reaction buffer with 0.8 µg UFM1ΔC2, UFM1ΔC2^R81C^ or UFM1^ΔGly83^ and some of the following: 5 mM ATP, 0.08 (for UFC1-UFM1 thioester formation assay) or 0.8 µg (for UBA5-UFM1 thioester formation assay) UBA5 and 0.8 µg UFC1, UFC1^R23Q^ or UFC1^T106I^. Reactions were incubated for 5 min at 25°C and stopped by the addition of NuPAGE® LDS Sample Buffer lacking reducing agent, followed by a 10-min incubation at 37°C, NuPAGE® (4–12% acrylamide gradient) and Coomassie brilliant blue staining. Data shown are representative of three separate experiments.

### Assessment of apoptosis in response to endoplasmic reticulum stress

HeLa or SH-SY5Y cells were plated into six-well plates at 7.5 × 10^5^ cells/ml. After 18 h they were transfected with either 2 µg GFP, 1 µg *UFM1*WT/1 µg GFP or 1 µg *UFM1*R81C/1 µg GFP using FuGENE® HD or X-tremeGENE™ HP, respectively according to the manufacturer’s protocols. Media was changed after 24 h and tunicamycin added after 48 h. For endoplasmic reticulum stress marker analysis, cells were incubated with 5 µg/ml tunicamycin for 8 h. mRNA was extracted from cells using the RNeasy® Plus Mini Kit (Qiagen) according to the manufacturer’s instructions. The concentration of the mRNA was calculated and 1 µg converted to cDNA using the qScript™ cDNA synthesis kit (Quanta Biosciences). Levels of *HSPA5*, *DDIT3* and *GAPDH* were assayed using pre-designed TaqMan® Gene Expression Assays (ThermoFisher Scientific). The level of gene expression was normalized to that of *GAPDH* for each sample assessed. For assessment of apoptosis in response to endoplasmic reticulum stress, cells were incubated in different concentrations of tunicamycin for 48 h. They were then, trypsinized and stained with Annexin V, Alexa Fluor® 647 conjugate (ThermoFisher Scientific) and DAPI to assess early and late stages of apoptosis, respectively. Cells were analysed on a BD LSR Fortessa™ flow cytometer, selecting only those expressing GFP. Data were analysed using FlowJo software, and cells expressing either DAPI and/or Annexin V were considered apoptotic.

## Results

### Identification of novel severe early infantile encephalopathy phenotypes

**Figure 1:**
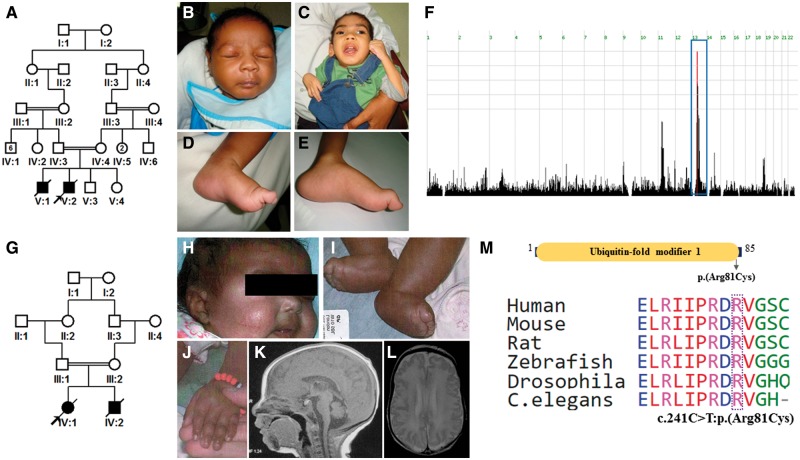
**UFM1-related clinical phenotype.** (**A**) Pedigree of Family 1 with *UFM1* mutation. (**B** and **C**) Images from Family 1 exhibiting lack of major facial dysmorphism. (**D** and **E**) Pes cavus as a result of abnormal tone in Patients 10DG0945 and 10DG0946, respectively (Family 1). (**F**) Genome-wide homozygosity mapping revealed a single critical locus. Blue box indicates haplotype (Chr13:36292810-42302880) identical by descent. (**G**) Pedigree of Family 2 with *UFM1* mutation. (**H**) Facial image of Patient UK1 (Family 2) showing full cheeks. (**I** and **J**) Peripheral oedema of Patients UK1 and UK2 (Family 2). (**K** and **L**) Brain MRI of Patient UK1 showing cerebellar hypoplasia and thin corpus callosum and frontal cortical polymicrogyria. (**M**) Schematic diagram of UFM1 protein representing ubiquitin-fold modifier 1 domain with the mutation indicated. The Arg81 at the mutation site is highly conserved from humans to *C. elegans*.

Two Sudanese families were recruited independently by C.G.W. and F.S.A., each with two children presenting with the core phenotype of profound global developmental delay, failure to thrive, progressive microcephaly and refractive epilepsy. Salient clinical features include subtle facial dysmorphism, severe axial hypotonia and appendicular hypertonia. Available brain imaging revealed dysmyelination and volume loss. Hypsarrhythmia was documented on EEG. The severity of the presentation is evidenced by the premature death of two of the four patients at ages 9 months and 8.5 years, respectively (Table 1, Fig. 1 and Supplementary Tables 1 and 2).

**Table 1:** Summary of the clinical features of patients with *UFM1* and *UFC1* mutations

ID	12DG0178	12DG1577	14DG0050	16DG1614	MDL-17-3196	MDL-17-3892	17DG0828	ID76366	UK1	UK2	10DG0945	10DG0946
Gene	*UFC1*	*UFC1*	*UFC1*	*UFC1*	*UFC1*	*UFC1*	*UFC1*	*UFC1*	*UFM1*	*UFM1*	*UFM1*	*UFM1*
Mutation	c.317C>T p.(Thr106Ile)	c.317C>T p.(Thr106Ile)	c.317C>T p.(Thr106Ile)	c.317C>T p.(Thr106Ile)	c.317C>T p.(Thr106Ile)	c.317C>T p.(Thr106Ile)	c.317C>T p.(Thr106Ile)	c.68G>A p.(Arg23Gln)	c.241C>T p.(Arg81Cys)	c.241C>T p.(Arg81Cys)	c.241C>T p.(Arg81Cys)	c.241C>T p.(Arg81Cys)
Age	16 years	23 years	3 years	5 years	5 years	31 months	8 years	4 years	13 months	13 months	2 years	1 year
Gender	F	F	F	F	F	F	F	M	F	M	M	M
Microcephaly**^a^**	+	+	-	+	+	+	+	+	+	+	+	+
Short Stature	+	+	-	+	+	+	+	+	+	+	+	+
Underweight	+	+	+	+	+	+	+	+	+	+	+	+
GDD	+	+	+	+	+	+	+	+	+	+	+	+
Seizures	+	-	-	+	+	-	-	+	+	+	+	+

GDD = global developmental delay; F = female; M = male.

^a^Microcephaly was always secondary i.e. postnatal.

**Figure 2:**
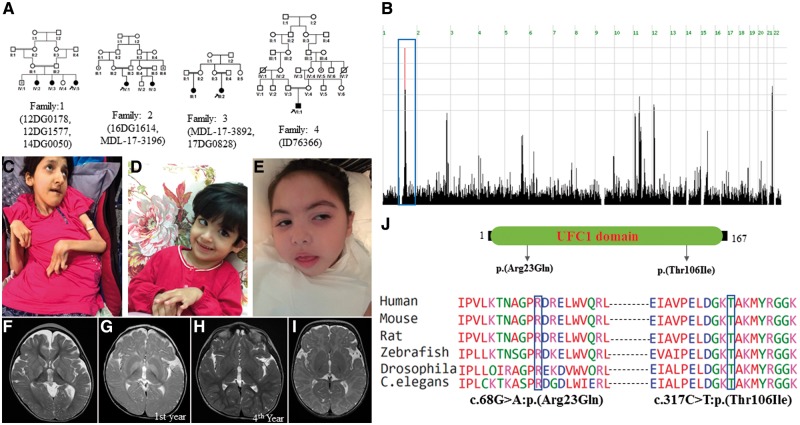
**UFC1-related clinical phenotype.** (**A**) Pedigrees of the families with *UFC1* mutation. (**B**) Genome-wide homozygosity plot highlighting a single critical locus from Families 1–3, who harbour the p.(Thr106Ile) mutation. Blue box indicates haplotype (Chr1:158957700-162094700) identical by descent. (**C**) Clinical images showing multiple joint contractures and emaciation (Patient 17DG0828). (**D** and **E**) Clinical images of younger patients suggest a progressive nature of emaciation (Patients 14DG0050 and 16DG1614, respectively). (**F**) Brain MRI of Patient 16DG1614 showing bilateral hyperintense signals in the basal ganglia. (**G** and **H**) Brain MRI of Patient MDL-17-3196 showing bilateral hyperintense signals in the basal ganglia in the first year that resolved in a repeated brain MRI in the fourth year of age. (**I**) Brain MRI of Patient ID76366 showing markedly delayed myelination. (**J**) Schematic diagram of UFC1 protein showing UFC1 domain and mutation site. Mutations Arg23Gln and Thr106Ile are highly conserved from humans to *C. elegans*.

Independently, we encountered a highly similar phenotype in three Saudi families and one Swiss family with eight affected members (one of which was previously described, albeit briefly) (Anazi *et al.*, 2016). They all presented with severe early infantile encephalopathy, progressive microcephaly, axial hypotonia, appendicular hypertonia and refractory epilepsy. Although brain MRI findings were largely non-specific, some had evidence of basal ganglia involvement (Table 1, Fig. 2 and Supplementary Tables 1 and 2). Phenotypic comparison of our patients with *UFM1* and *UFC1* mutations to previously reported patients with *UFM1* and *UBA5* mutations can be found in Table 2.

**Table 2:** Comparison of phenotype between this study patients with *UFM1* and *UFC1* mutations and those previously reported with mutations in *UFM1* and *UBA5*

	Comparison between UFM1-UBA5-UFC1 pathway reported cases			
	Current cohort		*UFM1*-related cases	*UBA5*-related cases
Gene	*UFC1*	*UFM1*	*UFM1*(PubMed: 28931644)	*UBA5*
				(PubMed: 27545681, 27545674, 28965491)
Mutation	c.317C>T; c.68G>A	c.241C>T	c.-273_-271delTCA	c.1111G>A
				c.904C>T
				c.971_972insC
				c.778G>A
				c.1165G>T
				c.169A>G
				c.503G>A
				c.164G>A
				c.684G > A
Number of cases	8	4	16	19
Failure to thrive	100% (8/8)	100% (4/4)	63% (10/16)	89% (8/9)
Short stature	88% (7/8)	100% (4/4)	75% (12/16)	77% (10/13)
Microcephaly	88% (7/8)	100% (4/4)	100% (16/16)	100% (18/18)
Global developmental delay	100% (8/8)	100% (4/4)	100% (16/16)	100% (19/19)
Seizures	50% (4/8)	100% (4/4)	75% (12/16)	84% (16/19)
Brain MRI				
Basal ganglia abnormality	33% (2/6)	0% (0/4)	100% (16/16)	0% (0/17)
Delayed CNS myelination	17% (1/6)	75% (3/4)	100% (16/16)	24% (4/17)
Cerebellar hypoplasia	0% (0/6)	75% (3/4)	81% (13/16)	24% (4/17)
Mortality	0% (0/8)	100% (4/4)	56% (9/16)	24% (4/19)

### *UFM1* and *UFC1* define novel loci for severe infantile encephalopathy with progressive microcephaly

Although the two Sudanese families are not known to be related, they originate from the same village in Sudan. Indeed, autozygome analysis revealed a single shared autozygous interval (Chr13:36292810-42302880) between the affected members with the same ancestral haplotype, strongly supporting a recessive founder mutation. Exome sequencing of the index in each of the two families revealed the same sole novel homozygous variant within this interval: *UFM1*: NM_016617.3:c.241C>T:p.(Arg81Cys) (Fig. 1).

Similarly, genotyping of the Saudi families with a similar encephalopathy phenotype revealed that they all share the same ancestral haplotype (Chr1:158957700-162094700), again supporting a recessive founder mutation (Fig. 2). Indeed, exome sequencing on four of the seven patients revealed a single novel homozygous variant within the critical locus: *UFC1*: NM_016406.3:c.317C>T:p.(Thr106Ile), which segregated with the phenotype in all four families as revealed by targeted Sanger sequencing. Exome sequencing on a Swiss patient revealed a novel homozygous variant in *UFC1*: NM_016406.3:c.68G>A:p.(Arg23Gln) inherited from the heterozygous parents. Mutations at Thr106 and Arg23 involve highly conserved residues (Fig. 2).

### Hypomorphic effect of *UFM1* mutation on the UFM1-system

**Figure 3:**
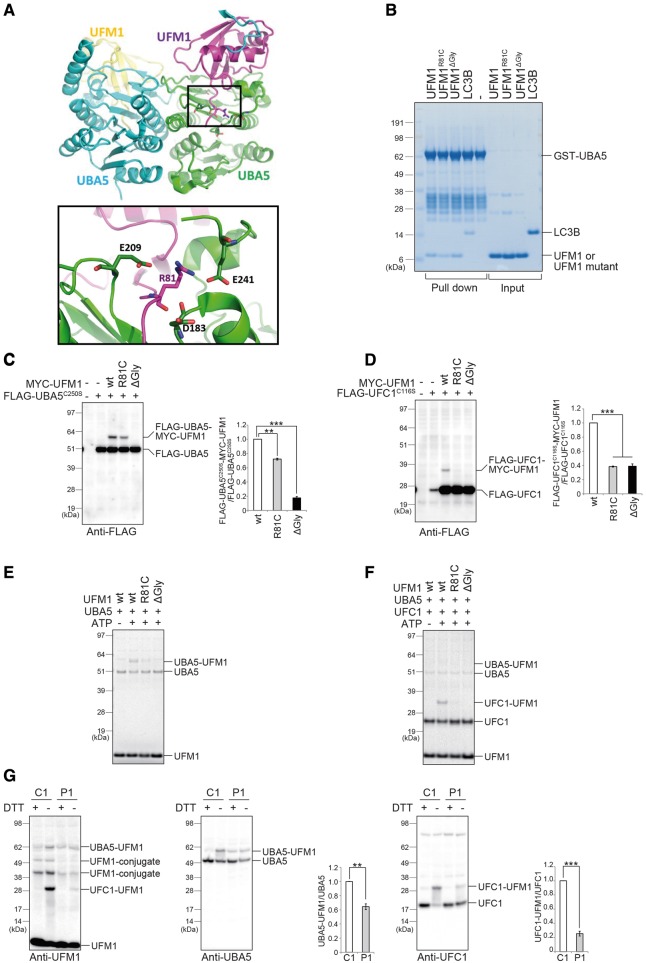
**Hypomorphic effect of the UFM1 mutation on the UFM system.** (**A**) Molecular basis for the effect of the UFM1^R81C^ mutation. *Top*: Crystal structure of the heterodimeric complex between UFM1 (magenta and yellow) and UBA5 (cyan and green), taken from PDB id 5IAA. *Bottom:* Magnification of the boxed region. The view is tilted horizontally by ∼30° compared to the *top* panel. For clarity, only side chains of Arg^81^ and of Arg^81^-interacting residues are shown. (**B**) *In vitro* pull-down assay. Pull-down assay with GST-UBA5 and UFM1, UFM1 mutants or LC3B. GST-UBA5 conjugated with Glutathione Sepharose® 4B was incubated with purified recombinant UFM1, UFM1 mutants or LC3B. LC3B is known to interact with UBA5. The pulled-down complexes were subjected to NuPAGE® (4–12% acrylamide gradient) and Coomassie brilliant blue staining. GST-UBA5, LC3B, UFM1 and UFM1 mutants are indicated. (**C** and **D**) Immunoblot assay. Indicated constructs (0.1 µg for UBA5^C250S^, 0.5 µg for UFC1^C116S^, and 2 µg for UFM1 or mutants) were expressed in *UFM1*-deficient HEK293T cells. Twenty-four hours after transfection, the cell lysates were subjected to immunoblot analysis with anti-FLAG antibody. Bar graphs indicate the quantitative densitometric analyses of FLAG-UBA5-MYC-UFM1 and FLAG-UFC1-MYC-UFM1 intermediates relative to free FLAG-UBA5 and FLAG-UFC1, respectively. Statistical analyses were performed using the unpaired *t*-test (Welch test). The data represent the means ± SE of three separate experiments. **P < 0.01 and ***P < 0.001. (**E** and **F**) *In vitro* thioester formation assay of UFM1 by UBA5 (**E**) and of UFM1 by UFC1 (**F**). The assay was conducted as described in the ‘Materials and methods’ section. Data shown are representative of three separate experiments. (**G**) Immunoblot analysis in case (P1: 10DG0945, Individual V1 in Fig. 1A) and control (C1: a healthy Sudanese young female) lymphoblasts. Reducing (DTT plus) and non-reducing (DTT minus) samples were prepared from lymphoblasts and subjected to immunoblot analysis for UFM1 (*left*), UBA5 (*middle*), and UFC1 (*right*). We used a hand-made anti-UFM1 antibody in this experiment since the commercial antibody did not recognize UFM1^R81C^. Bar graphs indicate the quantitative densitometric analyses of UBA5-UFM1 and UFC1-UFM1 intermediates relative to free UBA5 and UFC1, respectively. Statistical analysis was performed using the unpaired *t*-test (Welch test). The data represent the means ± SE of three separate experiments. **P < 0.01 and ***P < 0.001.

The *UFM1* variant is predicted pathogenic by PolyPhen (0.538/possibly damaging), SIFT (0/ deleterious) and CADD (35), as are the *UFC1* variants c.317C>T:p.(Thr106Ile) [PolyPhen (0.998/probably damaging), SIFT (0/deleterious), CADD (32)] and c.68G>A:p.(Arg23Gln) [PolyPhen (0.901/probably damaging), SIFT (0.02/deleterious) and CADD (35)]. We therefore carried out experiments to assess if and in which way these mutations affect ufmylation. The C-terminal tail of UFM1 (residues 79–83) is essential for its adenylation by the E1 enzyme UBA5 and subsequent thioester formation with UBA5 (Oweis *et al.*, 2016). The crystallographic structure of UBA5 in the complex with UFM1 (PDB ID 5IAA) shows that the tail region, encompassing Arg^81^_,_ directly binds to UBA5 (Fig. 3A). Since Arg^81^ interacts with the negatively charged residues Glu^241^, Glu^209^ and Asp^183^ of UBA5 (Fig. 3A). The substitution of Arg^81^ with a shorter and hydrophobic Cys is expected to markedly reduce the strength of the interaction between UFM1 and UBA5. Accordingly, an *in vitro* pull-down assay revealed that the mutant has a lower binding affinity to UBA5 than wild-type UFM1 (Fig. 3B). Next, we investigated whether UBA5 is able to activate UFM1^R81C^. To do this, we used a UBA5-mutant whose active site, cysteine (Cys^250^) was substituted with a serine (termed UBA5^C250S^). When the cysteine residue at the active site of E1 and E2 enzymes is replaced by a serine, an *O*-ester bond instead of a thioester bond is formed with its respective modifier proteins, which become stable even under reducing conditions (Komatsu *et al.*, 2004). To exclude an effect of endogenous UFM1, we generated *UFM1*-deficient HEK293T cells (Supplementary Fig. 1A). As expected, when we co-expressed FLAG-UBA5^C250S^ and wild-type MYC-UFM1 into *UFM1*-deficient HEK293T cells, MYC-UFM1 formed a stable intermediate with FLAG-UBA5^C250S^ (Fig. 3C). Such intermediate formation was completely abrogated when UBA5^C250S^ was co-expressed with MYC-UFM1^ΔGly83^, whose Gly^83^ essential for the adenylation and activation through UBA5 is deleted (Fig. 3C). Though we still observed the intermediate in the case of MYC-UFM1^R81C^, its level was only ∼75% of that of wild-type UFM1 (Fig. 3C).

The defective activation of UFM1^R81C^ by the UBA5 E1 enzyme is expected to also hamper the subsequent step, namely the formation of the intermediate with its cognate E2-like enzyme UFC1. Therefore, we investigated whether UFM1^R81C^ forms an intermediate with UFC1 in cells. When wild-type MYC-UFM1 was expressed together with FLAG-UFC1^C116S^ in which the active site, cysteine (Cys^116^), was substituted with serine, we clearly detected the intermediate of UFC1^C116S^ with MYC-UFM1 (Fig. 3D). Conversely, the formation of the UFM1-UFC1 intermediate was almost abolished when MYC-UFM1^R81C^ or MYC-UFM1^ΔGly83^ were expressed together with UFC1^C116S^ (Fig. 3D). In good agreement with those analyses, an *in vitro* thioester formation assay revealed that while wild-type UFM1 forms an intermediate with UBA5, UFM1^R81C^ slightly had the ability to form the intermediate (Fig. 3E). We also observed that UFM1^R81C^ is hardly transferred to UFC1 (Fig. 3F). In the next series of experiments, we tested the effect of the UFM1^R81C^ mutation on the intermediate formation with endogenous UBA5 and UFC1 as well as UFM1-conjugation, using lymphoblasts derived from an affected individual. Expression of free UFM1 protein in lymphoblasts from an affected individual was comparable to control cells (Fig. 3G, left panel). By immunoblot analysis with non-reducing samples, we detected the intermediates of UFM1-UBA5 and of UFM1-UFC1. As predicted, the level of UFM1-UBA5 intermediates was significantly lower in lymphoblasts of affected individual compared to control cells (Fig. 3G, middle panel). Likewise, the formation of the UFM1-UFC1 intermediate markedly declined (Fig. 3G, right panel). We also found that the level of two UFM1-conjugates with cellular proteins in patient-derived lymphoblasts was significantly reduced compared to control lymphoblasts (Fig. 3G, left panel). Taken together, our data show that UFM1^R81C^ has a hypomorphic effect on the UFM1-system and provide a plausible mechanistic explanation.

### Hypomorphic effect of *UFC1* mutations on the UFM1-system

**Figure 4:**
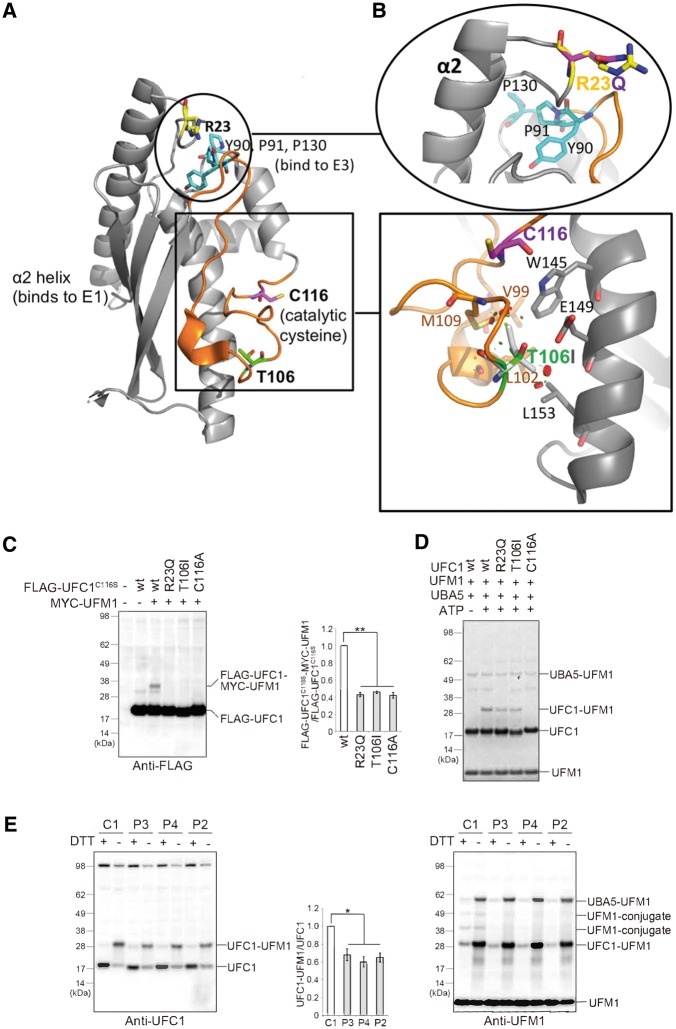
**Hypomorphic effect of UFC1 mutants on the UFM system.** (**A**) Localization of the key binding sites on UFC1. Binding sites for E1 and E3 are indicated. Boxed region shows the active site, and the coiled region for binding site to UFM1 is coloured in orange. The mutated residues Arg^23^ and Thr^106^ are highlighted in yellow and green, respectively. Encircled region shows the proximity of R23 with regions involved in binding to E1 (helix α2) and E3 (Tyr^90^, Pro^91^, Pro^130^; cyan). (**B**) *Bottom*: Magnification of Thr^106^. Residues involved in hydrophobic or polar interactions with Thr^106^ are shown as stick models. Colouring as in **A**, except for the mutant Ile^106^, which is shown as light grey stick model, with positions of minor clashes indicated as discs. *Top:* Magnification of Arg^23^. Colouring as in **A**, except for the mutant Gln^23^, which is shown as magenta stick model. (**C**) Immunoblot assay. Indicated constructs (0.5 μg for UFC1^C116S^, UFC1^C116S/T106I^ or UFC1^C116S/R23Q^ and 2 μg for UFM1) were expressed in UFC1-deficient HEK293T cells. Twenty-four hours after transfection, the cell lysates were subjected to immunoblot analysis with indicated antibodies. Bar graphs indicate the quantitative densitometric analyses of FLAG-UFC1-MYC-UFM1 intermediates relative to free FLAG-UFC1. Statistical analyses were performed using the unpaired *t*-test (Welch test). The data represent the means ± SE of three separate experiments. *P < 0.05. (**D**) *In vitro* thioester formation assay of UFM1 by UFC1. The assay was conducted as described in the ‘Materials and methods’ section. Data shown are representative of three separate experiments. (**E**) Immunoblot analysis in case (P2, P3, P4: V:1) and control (C1: a healthy Sudanese young females) lymphoblasts. Reducing (DTT plus) and non-reducing (DTT minus) samples were prepared from lymphoblasts and subjected to immunoblot analysis for UFC1 (*left*), and UFM1 (*right*). Bar graph indicates the quantitative densitometric analysis of UFC1-UFM1 intermediates relative to free UFC1. Statistical analysis was performed using the unpaired *t*-test (Welch test). The data represent the means ± SE of three separate experiments. **P < 0.01.

The UFC1 structure consists of the catalytic core domain conserved in all E2-like enzymes and an additional N-terminal helix (Mizushima *et al.*, 2007). Thr^106^ on the UFC1 structure is on the opposite site of the proposed UBA5 binding site (involving helix α2) (Liu *et al.*, 2009), suggesting that mutation of this residue does not affect the interaction with UBA5. Indeed, an *in vitro* pull-down assay showed that the Thr106Ile mutation does not have any effect on binding to UBA5 (Supplementary Fig. 2). Thr^106^ is located in a coiled region close to the catalytic Cys^116^, and within the site mapped to be required for interactions with UFM1 (Liu *et al.*, 2009) (orange segment in Fig. 4A). In the crystal structure of unliganded UFC1, Thr^106^ interacts with the hydrophobic core of the protein and is mostly excluded from the solvent (Fig. 4B). Replacing the polar threonine with the slightly larger and completely hydrophobic isoleucine is expected to change the anchoring, stereochemistry and dynamics of this region, affecting the intermediate formation of UFM1 with UFC1. Similarly, Arg^23^ is close (∼10 Å) to the residues Tyr^90^, Pro^91^ and Pro^130^ that form a substructure and is potentially involved in E3-binding (Liu *et al.*, 2009) (Fig. 4A). Arg^23^ is also near to the helix α2, which is involved in binding to E1 (Mizushima *et al.*, 2007). Consequently, the substitution of the positively charged Arg^23^ with a shorter and uncharged glutamine may affect binding of UFC1 to E1 or E3 (Fig. 4B). Therefore, we tested whether the UFC1^T106I^ and UFC1^R23Q^ variants impact on the ufmylation. To do this, HEK293T cells deleting *UFC1* were generated (Supplementary Fig. 1B), and MYC-UFM1 together with FLAG-UFC1^C116S^, FLAG-UFC1^T106I/C116S^ or FLAG-UFC1^R23Q/C116S^ was expressed in the *UFC1*-deficient HEK293T cells. While an UFC1-UFM1 intermediate was clearly detected in FLAG-UFC1^C116S^*-*expressing *UFC1*-deficient HEK293T cells, only a faint intermediate formation was observed in the case of FLAG-UFC1^T106I/C116S^ and FLAG-UFC1^R23Q/C116S^ (Fig. 4C). Our *in vitro* thioester formation assay also showed that while wild-type UFC1 formed an intermediate with UFM1, UFC1^T106I^ and UFC1^R23Q^ substantially reduced this ability (Fig. 4D). In lymphoblasts isolated from three affected individuals with UFC1^T106I^, the intermediate formation with endogenous UFM1 was significantly suppressed in comparison with that in control lymphoblasts (Fig. 4E, left panel). Similar to the case of UFM1^R81C^, the UFM1-conjugate formation with cellular proteins was impaired in patient-derived lymphoblasts (Fig. 4E, right panel). Taken together, we concluded that although the mutations UFM1^R81C^ and UFC1^T106I^ UFC1^R23Q^ affect different proteins, they all impact ufmylation. Hence our analysis explains the similar patient phenotype by showing that these mutations cause phenotypically similar hypomorphic effects on the UFM1-system.

### Mutation in UFM1 does not affect endoplasmic reticulum stress-mediated apoptosis

Studies have reported that the pathogenesis of ufmylation-related encephalopathy is associated with endoplasmic reticulum stress-mediated apoptosis. Thus we tested UFM1 mutation (p.R81C) effect on endoplasmic reticulum stress. We found no significant impact of *UFM1* wild-type or mutant expression on the induction of endoplasmic reticulum stress markers CHOP *(DDIT3)* and BIP *(HSPA5)* in either HeLa or SH-SY5Y cells, after 8 h treatment with 5 µg/ml tunicamycin (Supplementary Fig. 3). Neither did we note appreciable difference in endoplasmic reticulum stress-induced apoptosis in cells expressing *UFM1* wild-type or *UFM1* p.R81C, after 48 h incubation in tunicamycin at various concentrations.

## Discussion

Our understanding of the pathogenesis of infantile encephalopathy at the molecular level has greatly expanded in recent years due in large part to the rapidly growing use of genomics to diagnose and classify this highly heterogeneous group of disorders. The remarkable diversity and breadth of implicated molecular pathways are consistent with the highly complex nature of the brain (Hamdan *et al.*, 2017). Indeed, the observation that the majority of these conditions are not associated with significant systemic findings, further supports the notion that brain vulnerability is inherent to its complexity. This is especially remarkable when one considers the fundamental and ubiquitous nature of the many biological processes that are impaired in the various genetic forms of infantile encephalopathy, including post-translational modification (Alazami *et al.*, 2015; Anazi *et al.*, 2016, 2017).

Ufmylation is a highly conserved post-translational modification with each of its components having a corresponding orthologue in all multicellular organisms. In this study, we show that this pathway is critically required for normal brain development and function in humans consistent with suggestive data from model organisms. For example, the fruitfly models of UBA5 and UFM1 deficiency display increased mortality, locomotive defects, and abnormal neuromuscular junctions (Duan *et al.*, 2016). Similarly, deficiency of the UBA5 orthologue in *Caenorhabditis elegans* results in increased susceptibility to induced seizures and pharynx grinder paralysis as well as abnormal sensorial behaviour (Colin *et al.*, 2016). Impaired motility and seizure-like activity were also observed in zebrafish *uba5* morphants (Colin *et al.*, 2016). More relevant to the phenotype of the patients we described in this study is our recently published brain-specific conditional knockout of *Ufm1* under the nestin promoter (Muona *et al.*, 2016), which allowed us to directly observe the brain pathology associated with impaired ufmylation *in vivo*. Although these mice appeared normal at birth, they uniformly died in the first day after birth. Histopathological examination revealed that their brains were microcephalic with evidence of increased apoptosis, consistent with the proposed role of ufmylation in neuronal development and survival (Muona *et al.*, 2016).

Similar to our previous work on *UBA5*-related severe infantile encephalopathy, we show that mutations in *UFM1* itself as well as in *UFC1*, encoding the sole E2 conjugating enzyme for UFM lead to widespread impairment of ufmylation. Our results show a reduction rather than abrogation of ufmylation with the activity of UFM1 and UFC1 mutants at 60–75% of their wild-type counterparts. A recently reported promoter mutation in *UFM1* in the context of infantile encephalopathy with or without epilepsy is similarly predicted to only reduce but not eliminate transcription of an otherwise normal transcript (Hamilton *et al.*, 2017). The clinical phenotype of previously reported patients with *UBA5* (Colin *et al.*, 2016; Duan *et al.*, 2016; Muona *et al.*, 2016) and *UFM1* (Hamilton *et al.*, 2017) mutations are similar to our *UFM1* and *UFC1* patients, particularly regarding failure to thrive, short stature, microcephaly, GDD, seizures, basal ganglia abnormality, delayed CNS myelination, and cerebellar hypoplasia (Table 2). These observations strongly argue for a minimum threshold required for embryonic viability, and this would be consistent with the mouse embryonic lethal phenotype observed in the complete knockout of *Uba5*, *Ufbp1*, *Ufl1* or *Ufm1* (M.K., unpublished data) (Tatsumi *et al.*, 2011; Cai *et al.*, 2015; Zhang *et al.*, 2015).

The pathogenesis of ufmylation-related encephalopathy remains unclear e.g. is the disease process a progressive apoptosis of selected neuron classes, or a neuronal dysfunction that becomes increasingly evident during development? The known localization of the ufmylation cascade and target proteins to the luminal side of the endoplasmic reticulum and the proposed role in regulating the unfolded protein response (UPR) and endoplasmic reticulum stress-mediated apoptosis, suggest a potential mechanistic link (Lemaire *et al.*, 2011; Zhang *et al.*, 2015; Ishimura *et al.*, 2017). Expanded endoplasmic reticulum network and increased endoplasmic reticulum volume in response to tunicamycin, both markers of endoplasmic reticulum stress, have indeed been observed in fibroblasts from patients with *UBA5* mutations (Colin *et al.*, 2016). Long-term endoplasmic reticulum stress driven by mutation of specific disease-related genes is known to cause various adult-onset neurodegenerative phenotypes by overwhelming the UPR and inducing apoptosis (Hetz and Saxena, 2017). While we found no evidence of *UFM1* wild-type or p.R81C overexpression affecting endoplasmic reticulum stress induction or apoptosis in response to tunicamycin (Supplementary Fig. 3), further work should serve to investigate the mechanisms by which defects in ufmylation lead to early-onset encephalopathy in humans. However, it may be that the ufmylation cascade, rather than a global effect on endoplasmic reticulum or UPR, could disrupt neuron function through effects on neural-essential proteins; the neuronal cell adhesion molecule (NCAM) interacts and co-localizes with UFC1, and CDK5 activity is controlled by CDK5RAP3, which aggregates with UFL1 and UFSP2 at the endoplasmic reticulum membrane (Homrich *et al.*, 2014).

In conclusion, our study suggests that impaired ufmylation leads to a recognizable syndrome of severe infantile encephalopathy and progressive microcephaly with or without epilepsy. Further studies are needed to discern the exact pathomechanism of ufmylation-related neurodevelopmental disorder, which may lead to possible therapies especially when one considers the hypomorphic nature of the observed mutations.

## Supplementary Material

Supplementary Data
