# Supplementary material for: Biallelic UFM1 and UFC1 mutations expand the essential role of ufmylation in brain development
Source: Brain. 2018 Jun 2;141(7):1934–45. doi: 10.1093/brain/awy135 (PMC6022668; doi:10.1093/brain/awy135)
Supplement: Supplementary Data [file awy135_suppl_data.zip › brain-2017-02432-File011.pdf]

Identification Number	CLINICAL SYNOPSIS
12DG0178<br>*UFC1*<br>(NM_016406.3:c.317C>T:p.(Thr106Ile))	A 16-year-old girl who was born at full term via NSVD to a G4P3 mother following an uneventful pregnancy with a birth weight of 3.5 kg. She was found to be floppy since birth, and she had a delay in acquiring milestones. She started to have spasticity at four years of age and seizures at 13 years of age. Developmentally, she has a profound global developmental delay. She is a wheelchair bound, and she cannot control her head, transfer or talk. She can hold objects momentarily, and her speech is limited to babbling. Her parents are a first-degree consanguineous couple, and they have three affected children and four healthy children. Physical examination revealed hirsutism, spasticity, hyperreflexia and mild intention tremor. Her growth parameters at 16 years of age were weight 22.2 kg (<3rd percentile) and head circumference 51 cm (< 3rd percentile). Extensive investigations were within normal limits. Brain MRI showed white matter changes.
12DG1577<br>*UFC1*<br>(NM_016406.3:c.317C>T:p.(Thr106Ile))	A 23-year-old girl who was born to a G2P1 healthy mother. She is known to have a profound global developmental delay, spasticity and ataxia. On examinations, she is a wheelchair bound, and neurological examination revealed hypertonia that is more prominent in the lower limbs, hyperreflexia with a clonus, intention tremor and oculomotor apraxia. Her growth parameters at 19 years of age were weight 27.8 kg (< 3rd) and head circumference 50 cm (<3rd percentile). Brain MRI at 14 years of age was reported as normal.
14DG0050<br>*UFC1*<br>(NM_016406.3:c.317C>T:p.(Thr106Ile))	A 3-year-old girl who was born at 40 weeks gestation via NSVD to a G7P6 healthy mother. Pregnancy was uneventful, and Apgar scores were 7 and 8 at 1 and 5 minutes, respectively. Growth parameters at birth were weight 4.01 kg, length 51 cm and head circumference 34 cm, and she was admitted to NICU after birth due to hypoglycemia and discharged on the fourth day. Since birth, she has been noted to be floppy and had abnormal eye alignment. Developmentally, she has always been delayed. She rolled over at the age of 5 months, sat with support at 9 months, but said baba and mama at 12 months. Growth parameters at 12 months were weight 6.6 kg (< 3rd percentile) height 69 cm (10th-25th percentile) and head circumference 42.5 cm (10th-25th percentile). Her neurological examination revealed appendicular hypertonia, axial hypotonia and hyperreflexia in the lower limbs. Her ophthalmological assessment was significant for hypertropia, esotropia and

	hypermetropia, but other examinations were within normal limits. A brain MRI that was done in the first months of life was reported to be normal.
16DG1614<br>*UFC1*<br>(NM_016406.3:c.317C>T:p.(Thr106Ile))	A 5-year-old girl who was born at 32 weeks gestation via vaginal delivery. Pregnancy was complicated by preterm premature rupture of membranes 2 days before delivery. Birth weight was 1.5 kg, and Apgar scores were 8 and 9 at 1 and 5 minutes, respectively. She was admitted to the NICU where she was started on mechanical ventilation for a week, and then she was weaned from the ventilator and remained for four weeks in the NICU. After discharge, she had multiple respiratory infections and seizures that led to multiple hospital admissions. Her seizures are poorly controlled. She is on GT tube for feeding. She also was noted to have severe GERD, and she underwent fundoplication procedure twice. At the age of 7 months, she started to have continuous abnormal movements in all extremities which began suddenly. Developmentally, she has a profound global developmental delay. She cannot walk, roll over, crawl, talk or say any word. Her parents are first-degree cousins, and there is a family history of intellectual disability on the paternal side. On examination, she has no dysmorphic features, and all growth parameters are below the 3rd percentile. Her neurological examination was significant for has axial hypotonia and appendicular hypertonia with brisk reflexes. EEG showed bilateral centrotemporal spikes, more pronounced in the right hemisphere. Brain MRI showed diffuse white matter volume loss, more noticeable around the central sulcus. There was an abnormal high signal intensity symmetric within globus paladi and extending into the midbrain involving the sabstantia nigra and red nucleus bilaterally. There was an incidental finding of prominent cisterna magna.
MDL 17-3196<br>*UFC1*<br>(NM_016406.3:c.317C>T:p.(Thr106Ile))	A 5-year-old girl who was born at 37 weeks gestation via NSVD to a G5P4 31 years old healthy mother. Pregnancy was uneventful, and growth parameters at birth were weight 1.94 kg (<3rd percentile), length 43 cm (<3rd percentile) and head circumference 31 cm (on 10th percentile). Apgar scores were 9 and 9 at 1 and 5 minutes, respectively. She was admitted to the special care nursery at 2 hours of age due to low birth weight, and the mother asked for discharge on the 4th day of life against medical advice. After she was discharged from the hospital, she had poor feeding and was irritable most of the time. The first concern was noted at the age of one month, and it was spasticity and persistent crying. Echocardiogram at six months of age revealed a patent foramen ovale and small

	PDA. At the age of 8 months, she developed seizures with a semiology of infantile spasms (flexion of the neck and extension of the arms) and the EEG was consistent with hypsarrhythmia, but they resolved at the age of two years. She had recurrent respiratory infections, dental infections and urinary infections, and she was found to have bilateral vesicoureteral reflux grade 3. She also was found to have amblyopia, severe GERD and chronic constipation. Developmentally, she has a profound global developmental delay affecting all domains. She can sit with support, babble, grasp momentarily and respond to her name. Her parents are not consanguineous, but they are from the same tribe, and they have another three healthy children with no history of abortion or stillbirth. There is a family history of a similar condition in a cousin. On examination, her last growth parameters at five years of age were weight 9.6 kg (<3rd percentile), height 90 cm (<3rd percentile) and head circumference (<3rd percentile). Her dysmorphic features include sloping forehead, flat occiput, prominent nose, small mouth, upturn nostril, long eyelashes, microcephaly, micrognathia, pectus excavatum, muscle atrophy, synophrys and low set ears. Her neurological examination was significant for axial hypotonia, appendicular hypertonia, quadriplegia and extensor plantar responses. Other examinations including funduscopic evaluation were within normal limits. She was reported to be hypersensitive to sounds, but her hearing assessment was within normal limits. Pelvic X-ray showed bilateral coxa valga deformity. CBC, electrolytes, ammonia, CSF lactate, serum lactic acid, LDH, uric acid, CSF and plasma amino acids, urine organic acids, liver function tests, renal function tests, biotinidase, pyruvate, ACTH, cortisol, TSH and CGH microarray were within normal limits. Brain MRI at eight months of age was reported to be normal. Brain MRI three months later showed signal hyper intensity involving the globus pallidus bilaterally which appeared swollen with restricted diffusion. The degree of myelination was relatively delayed. A repeated brain MRI at four years of age showed resolution of the previous abnormal signal intensity within the globus pallidus with proper myelination.
MDL 17-3892<br>*UFC1*<br>(NM_016406.3:c.317C>T:p.(Thr106Ile))	A 31-month-old girl who was born at 37 weeks gestation via NSVD to a primigravida following an uneventful pregnancy. Her growth parameters at birth were weight 2.2 kg (10th-25th percentile), length 48 cm (50th-75th percentile) and head circumference 32.5 cm (25th-50th percentile), and she was discharged on the second day. The first concern

	was at four months of age when she was noted to have poor feeding, choking, floppiness and a stridor that was due to laryngomalacia. She also has a history of arching during sleep and feeding. She has a profound global developmental delay in all domains. She currently cannot roll over or sit, and she has no language development. However, she can grasp, smile and laugh. Her parents are a first-degree consanguineous couple, and there is a history of a similar condition in two cousins on the maternal side. Her examination is significant for axial hypotonia, appendicular hypertonia, pitting nails, hypertelorism, epicanthal folds, small ears, upturned nostrils, expressionless face and esotropia. Her growth parameters at 28 months of age were weight 10.1 kg (<3rd percentile), height 82 cm (<3rd percentile) and head circumference 45 cm (on the 3rd percentile). Newborn screening was normal.
17DG0828<br>*UFC1*<br>(NM_016406.3:c.317C>T:p.(Thr106Ile))	An 8-year-old girl with facial dysmorphia, microcephaly, intellectual disability, failure to thrive, simian crease and skeletal anomalies. She was born at full term via NSVD to a healthy mother following a pregnancy that was complicated by a first trimester bleeding with a birth weight of 3.5 kg. Developmentally, she had a profound global developmental delay. At 8 years of age, she was still unable to sit unsupported, stand or walk. Her parents are consanguineous with other healthy children. Her examination revealed facial dysmorphia, hypertonia, hyperreflexia, simian crease, microcephaly, spasticity, bilateral fixed flexion deformity of the knees, valgus feet, vertical talus and bilateral tight Achilles tendon. Growth parameters at 7 years were weight 10.1 kg (<3rd percentile, -4.7 SD), height 91 cm (<3rd percentile, -5.8 SD) and head circumference 45.2 cm (<3rd percentile, -5.1 SD). Head CT was normal, and spine X-rays showed mild scoliosis.
**ID76366**<br>*UFC1*<br>(NM_016406.3:c.68G>A:p.(Arg23Gln))	A 4-year-old Swiss boy who was born after an uneventful pregnancy with normal birth growth parameters, weight 3.2 kg [10-25th percentile], height 49 cm [5-10th percentile] and head circumference 34 cm [10th percentile]. He presented within the first days of life with hyperexcitability, trembling, pronounced hyperreflexia and myoclonus that was predominantly in the legs. At the age of 3 months, he developed pharmacoresistant serial infantile spasms. The EEG at that time showed tonic seizure patterns and hypsarrhythmia, and a brain MRI showed a markedly delayed myelination. Head sonography and lumbar puncture were normal. A month later, the patient presented with severe generalized dystonia, hypokinesis, spastic tetraparesis with opisthotonos and eye deviation, and

	secondary failure to thrive. At the age of 8 months, he underwent PEG-tube insertion, and his seizures worsened with multiple daily episodes of serial seizures and new myoclonic seizures as well as recurrent vomiting. His epilepsy remained pharmacoresistant to phenobarbital, vigabatrin, topiramate, clobazam, zonisamide and ketogenic diet. He was also found to have fragmented sleeping pattern with vomiting episodes. Developmentally, he has a profound developmental delay. At the age of 16 months he started babbling, and he was not able to grasp, sit, walk or talk. He is the only child of a distantly consanguineous Swiss couple. His physical examination revealed microcephaly, short stature, no visual fixation, a high, narrow palate, a submucous cleft palate, tented upper lip vermilion, full cheeks, hypoplasia of the midface, large earlobes, and short fingers. His growth parameters at 4 years of age were weight 12.9 kg (-2.1 SD), height 80 cm (-5.3 SD) and head circumference 45 cm (-3.6 SD).
10DG0945 and 10DG0946<br>***UFM1***<br>(NM_016617.3:c.241C>T:p.(Arg81Cys))	A 2-year-old deceased boy with global developmental delay. He was born at full term via emergency C-section due to fetal distress with a birth weight of 2.8 kg. He was admitted to NICU for two weeks and was mechanically ventilated for the first 48 hours. He had hypertonia and feeding difficulty that was managed by NGT feeding for 2 months. At the age of 2.5 months, he developed seizures with a semiology of clonic movements that were mainly on the right side. Developmentally, he had a global developmental delay, and he could not control his head, sit or reach objects at 2 years of age. His parents are a first-degree consanguineous couple with a family history of spinal muscular atrophy in a maternal male cousin. His growth parameters at 24 months of age were weight 5.4 kg, height 71 cm and head circumference 40 cm. His physical examination revealed large ears, poor body built, abdominal distention, appendicular hypertonia, central hypotonia, diminished reflexes and bilateral pes cavus. His ophthalmological assessment was normal, and the examination of other systems was normal. His nerve conduction was consistent with congenital peripheral neuropathy. Karyotyping, metabolic screening, VLCFA and L-acids were within normal limits. SMA was excluded by genetic testing. His brain MRI showed delayed white matter myelination, Dandy-Walker like cyst in the posterior fossa, hypoplastic cerebellum and hypoplastic corpus callosum.

	His younger deceased brother had a similar clinical course and clinical features. His brain MRI showed delayed white matter myelination. He died at the age of 1 year.
UK1 and UK2<br>***UFM1***<br>(NM_016617.3:c.241C>T:p.(Arg81Cys))	A 13-month-old deceased girl who had hypotonia and nystagmus at birth. Prenatal ultrasound at 30 weeks gestation showed cerebellar hypoplasia that was not present at 20 weeks. She had to be nasogastric tube fed after birth. Seizures commenced in the first month and continued through the first year, requiring changes of medication. Infantile spasms were diagnosed by three months, and an EEG at five months showed hypsarrhythmia. She often hyperventilated, but the cause of this was unclear. Thereafter, the fits became more difficult to control and were generalized as well as focal. She had facial features reminiscent of PEHO syndrome, as well as persisting mild edema of her hands and feet; and PEHO syndrome had been the working clinical diagnosis. She did not have optic atrophy but had little visual attention after 6 months of age with a diagnosis of cortical blindness. She passed away at 13 months of age, the consequence of her progressive cognitive and motor decline. Her parents were first-degree cousins originated from Ethiopia. Whilst having normal growth parameters at birth, she developed secondary microcephaly (< -4 SD) by 13 months of age, and was generally poorly grown with height and weight (< -2 SD). Brain MRI scanning at two months of age showed cerebellar hypoplasia and diffuse cortical hypomyelination.<br><br>Her brother had a very similar clinical course and clinical features, and fits were noted in the first week. He died at the age of 13 months.
