# Supplementary material for: Biallelic UFM1 and UFC1 mutations expand the essential role of ufmylation in brain development
Source: Brain. 2018 Jun 2;141(7):1934–45. doi: 10.1093/brain/awy135 (PMC6022668; doi:10.1093/brain/awy135)
Supplement: Supplementary Data [file awy135_suppl_data.zip › brain-2017-02432-File012.pdf]

Supplemental Table S2

Phenotype	**12DG0178**	**12DG1577**	**14DG0050**	**16DG1614**	**MDL-17-3196**	**MDL-17-3892**	**17DG0828**	**ID76366**	**UK1**	**UK2**	**10DG0945**	**10DG0946**
Gene	*UFC1*	*UFC1*	*UFC1*	*UFC1*	*UFC1*	*UFC1*	*UFC1*	*UFC1*	*UFM1*	*UFM1*	*UFM1*	*UFM1*
Mutation	c.317C>T:p.(Thr106Ile)	c.317C>T:p.(Thr106Ile)	c.317C>T:p.(Thr106Ile)	c.317C>T:p.(Thr106Ile)	c.317C>T:p.(Thr106Ile)	c.317C>T:p.(Thr106Ile)	c.317C>T:p.(Thr106Ile)	c.68G>A:p.(Arg23Gln)	c.241C>T:p.(Arg81Cys)	c.241C>T:p.(Arg81Cys)	c.241C>T:p.(Arg81Cys)	c.241C>T:p.(Arg81Cys)
Dysmorphic Features												
Sloping forehead	-	-	-	-	+	-	-	-	-	-	+	+
Flat occiput	-	-	-	-	+	-	NA	-	-	-	NA	NA
Nose	-	-	-	-	Prominent nose	-	Prominent nasal bridge	-	-	-	-	-
Narrow mouth	-	-	-	-	+	-	-	-	-	-	-	-
Anteverted nares	-	-	-	-	+	-	-	-	-	-	+	+
Long eyelashes	-	-	-	-	+	-	-	-	-	-	+	+
Micrognathia	-	-	-	-	+	-	-	-	+	+	+	+
High, narrow palate	-	-	-	-	-	-	-	+	-	-	NA	NA
Tented upper lip vermilion	-	-	-	-	-	-	-	+	-	-	+	+
Full cheeks	-	-	-	-	-	-	-	+	+	+	+	+
Hypoplasia of the midface	-	-	-	-	-	-	-	+	-	-	-	-
Large earlobes	-	-	-	-	-	-	-	+	-	-	+	+
Pectus excavatum	-	-	-	-	+	-	NA	-	-	-	NA	NA
Synophrys	-	-	-	-	+	-	-	-	-	-	-	-
Low-set ears	-	-	-	-	+	-	-	-	-	-	+	+
Microtia	-	-	-	-	-	+	-	-	-	-	-	-
Hypertelorism	-	-	-	-	-	+	-	-	-	-	-	-
Strabismus	-	-	+	-	-	+	+	-	-	-	+	+
Epicanthus	-	-	-	-	-	+	-	-	-	-	-	-
Hair	Hirsutism	-	-	-	-	-	Hirsutism	-	-	-	-	-
Neurological												
Infantile spasms	-	-	-	+	+	-	-	+	+	+	-	-
Reflexes					+ Extensor plantar responses	NA						
Appendicular Tone												
Axial Tone	—	NA	—	—	—	—	NA	—	—	—	—	—
Ataxia	-	+	-	-	-	-	-	-	-	-	-	-
Tremor	+	+	-	-	-	-	-	-	-	-	-	-
Generalized dystonia	-	-	-	-	-	-	-	+	-	-	-	-
Hypsarrhythmia	-	NA	NA	NA	+	NA	NA	+	+	+	NA	NA
GERD	-	-	-	+	+	-	-	+	-	-	-	-
Recurrent respiratory infections	-	-	-	+	+	-	-	+	-	+	-	-
MRI												
BG Involvement	-	-	-	+	+	NA	NA	-	-	-	-	-
Other MRI Findings	1. Abnormality of the cerebral white matter	-	-	1. Cerebral white matter atrophy	-	NA	NA	1. Delayed CNS myelination	1. Delayed CNS myelination 2. Cerebellar hypoplasia	1. Abnormality of the cerebral white matter. 2. Cerebellar hypoplasia	1. Delayed CNS myelination 2. Cerebellar hypoplasia	1. Delayed CNS myelination
				2. Enlarged cisterna magna								
Others		1.Oculomotor apraxia	1.Hypermetropia	1. EEG abnormality	1.vesicoureteral reflux 3	1.Laryngomalacia	1.Single transverse palmar crease	1.Short fingers	1.Optic atrophy	1. Micropenis	1.Abdominal distention	1.Abdominal distention
					2. Amblyopia	2. Mask-like facies	2.Knee flexion contracture	2.Fragmented sleeping pattern with vomiting episodes	2.Minor contractures of left fingers	2. Died at the age of 13 months	2. Pes cavus	2. Pes cavus
					3. Generalized amyotrophy	3. Nail pits	3. Elbow flexion contracture	3.Hyperexcitability	3. Died at of age 13 months	3. Peripheral edema	3. Congenital peripheral neuropathy	3. Congenital peripheral neuropathy
					4. Sound sensitivity		4. Achilles tendon contracture	4. EEG abnormality	4. Peripheral edema		4. Died at 2 years of age	4. Died at 1 year of age
					5. Patent ductus arteriosus		5. Scoliosis					
