# Supplementary material for: Biallelic UFM1 and UFC1 mutations expand the essential role of ufmylation in brain development
Source: Brain. 2018 Jun 2;141(7):1934–45. doi: 10.1093/brain/awy135 (PMC6022668; doi:10.1093/brain/awy135)
Supplement: Supplementary Data [file awy135_suppl_data.zip › brain-2017-02432-File015.pdf]

Supplemental Information

Supplemental Figure S1

Characterization of *UFM1* and *UFC1* knockout cells. (A and B) Immunoblot analysis. Cell lysates of *UFM1*-knockout (A) and *UFC1*-knockout (B) HEK293T cells were subjected to NuPAGE, followed by immunoblot analysis with indicated antibodies. Data shown are representative of three separate experiments.

Supplemental Figure S2

In vitro pull-down assay. Pull-down assay with GST-UBA5 and UFC1, $UFC1^{T106I}$ or Atg3. GST-UBA5 conjugated with glutathione-Sepharose 4B was incubated with purified recombinant UFC1, UFC1, $UFC1^{T106I}$ or Atg3. Atg3, which is an E2 enzyme for Atg8, was used as a negative control. The pulled-down complexes were subjected to NuPAGE (4–12% acrylamide gradient) and Coomassie brilliant blue staining. GST-UBA5, Atg3, UFC1 and $UFC1^{T106I}$ are indicated.

Supplemental Figure S3

Impact of *UFM1* on endoplasmic reticulum stress. Hela and SH-SY5y cells were treated with 5ug/ml Tunicamycin for 8 hours to induce endoplasmic reticulum stress. A) and C) represent induction of *DDIT3* in Hela and SH-SY5Y cells expressing GFP control, UFM Wild Type and UFM1 p.R81C. Note upregulation of gene expression after tunicamycin treatment but no significant difference in response between the three transfected conditions. B) and D) represent the induction of HSPA5 from the same experiments. E) and F) represent the percentage of live/apoptotic cells for UFM1 Wild Type and UFM1 p.R81C after 48 hour of endoplasmic reticulum stress induction by incubation with a series of concentrations of Tunicamycin.

Supplemental Table S1

Detailed clinical reports on all patients reported in this study

Supplemental Table S2

Detailed clinical comparison between all patients reported in this study in a tabular format.
