# Supplementary figures and images for: Biallelic UFM1 and UFC1 mutations expand the essential role of ufmylation in brain development
Source: Brain. 2018 Jun 2;141(7):1934–45. doi: 10.1093/brain/awy135 (PMC6022668; doi:10.1093/brain/awy135)

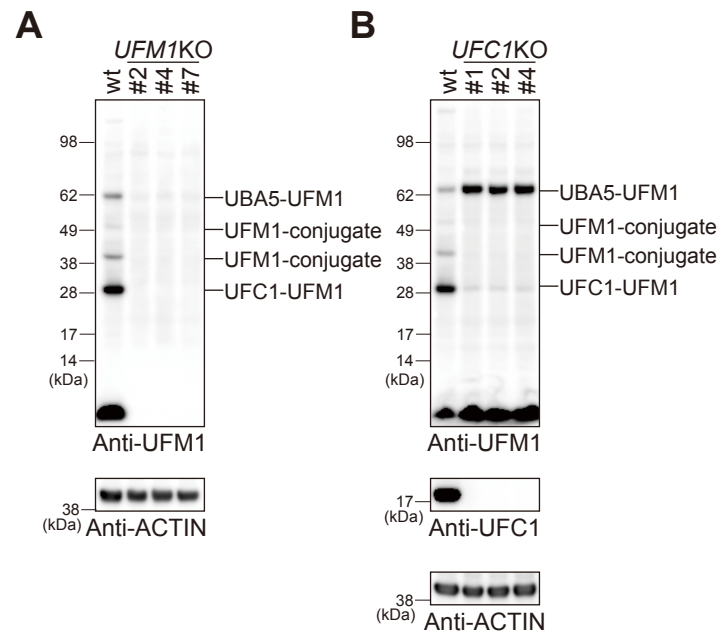

Supplementary Figure 1

Supplement: Supplementary Data [file awy135_suppl_data.zip › brain-2017-02432-File008.pdf]

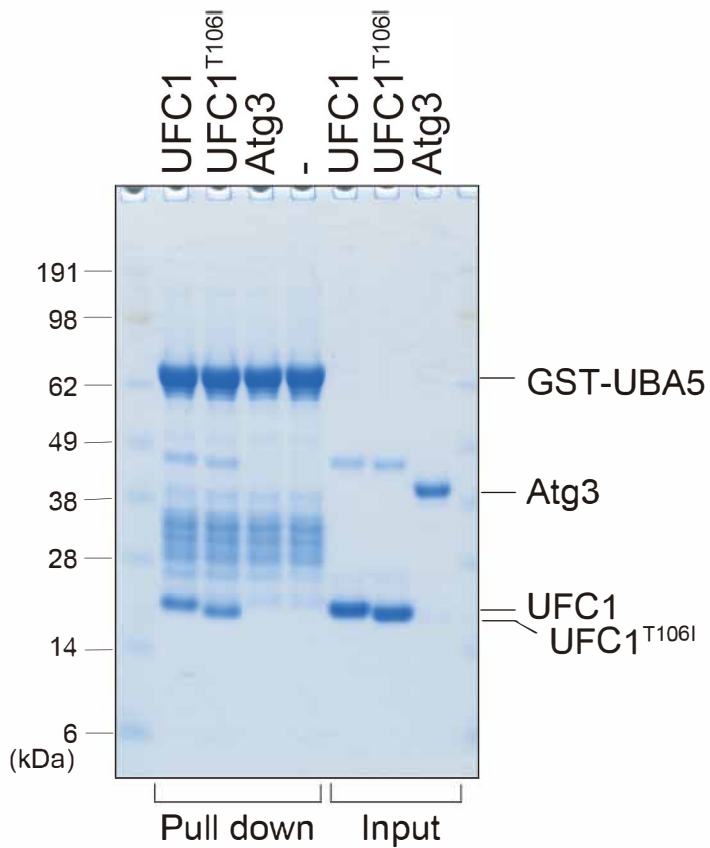

Supplementary Figure 2

Supplement: Supplementary Data [file awy135_suppl_data.zip › brain-2017-02432-File009.pdf]

Figure S3

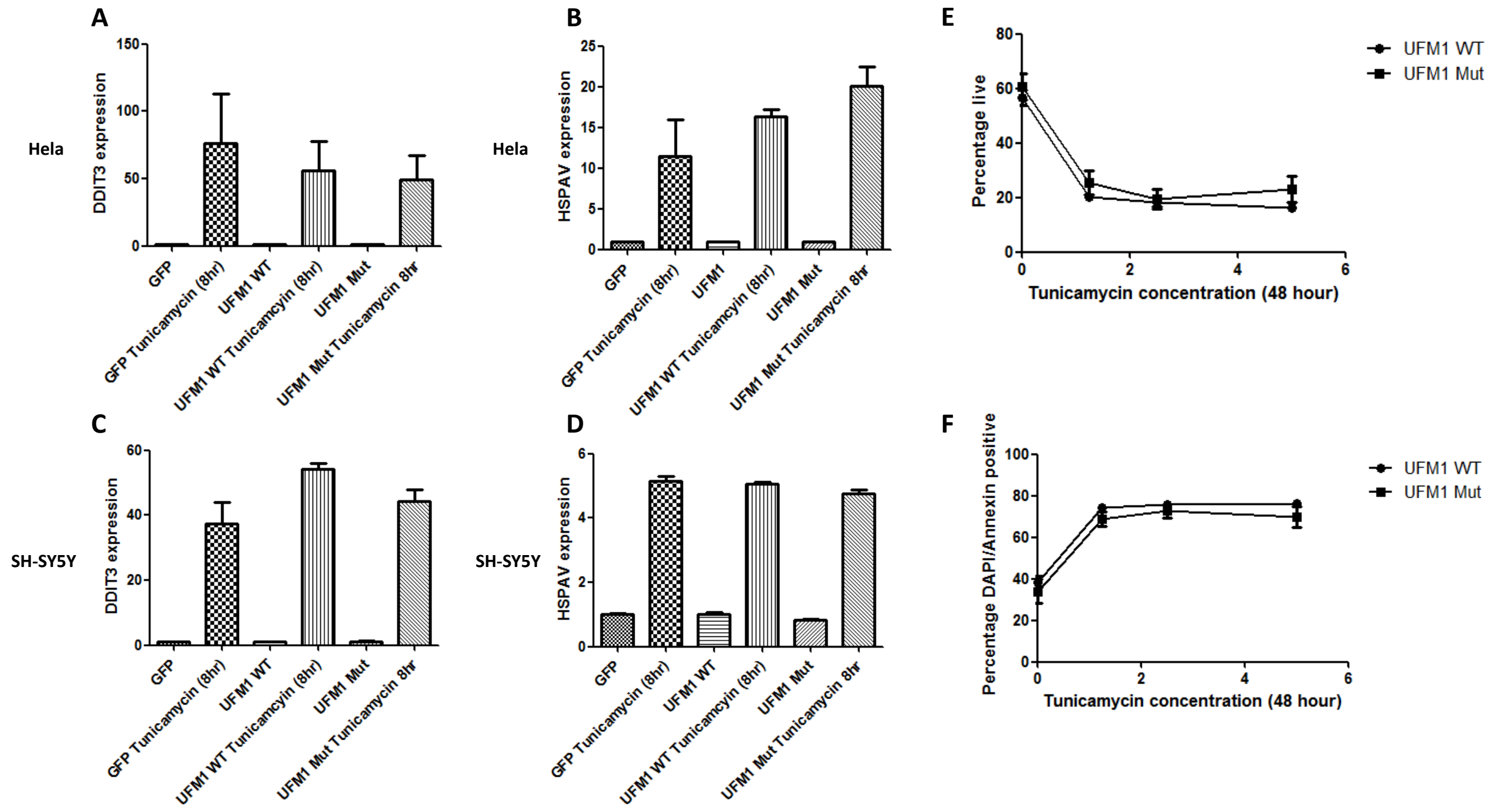

Supplement: Supplementary Data [file awy135_suppl_data.zip › brain-2017-02432-File010.pdf]
